# Self-reported visual function and in-depth swept-source optical coherence tomography features of cystoid macular edema in retinitis pigmentosa

**DOI:** 10.1186/s40942-024-00608-7

**Published:** 2024-11-22

**Authors:** Celso Costa, Carlos Nogueira, Mário Soares, Silvia Simão, Pedro Melo, Rufino Silva, Joaquim Murta, João Pedro Marques

**Affiliations:** 1 Ophthalmology Department, Unidade Local de Saúde de Coimbra (ULS Coimbra), Coimbra, 3004-561 Portugal; 2 Faculty of Medicine, University of Coimbra (FMUC), Coimbra, Portugal; 3 Clinical Academic Center of Coimbra (CACC), Coimbra, Portugal

**Keywords:** Inherited-retinal degenerations, Retinitis pigmentosa, Cystoid macular edema, Michigan retinal degeneration questionnaire

## Abstract

**Purpose:**

To evaluate self-reported visual function in retinitis pigmentosa (RP) patients with and without cystoid macular edema (CME) and to explore associations between cystoid spaces (CS), retinal morphometric parameters, and clinical data using swept-source optical coherence tomography (SS-OCT).

**Methods:**

In this cross-sectional case-control study (1:3) conducted at an Inherited Retinal Degenerations referral center in Portugal, RP patients with and without CME (matched for age and gender) completed the Michigan Retinal Degeneration Questionnaire (MRDQ) and underwent SS-OCT. Morphometric analysis, including ellipsoid zone area (EZA), was performed by two independent graders. In the CME group, detailed CS analysis was conducted. Correlations between clinical data - age, gender, best-corrected visual acuity (BCVA) - and MRDQ domains were evaluated.

**Results:**

The study included 23 RP patients with CME (60.87% male, mean age 44.65 ± 13.58 years) and 69 without CME (49.28% male, mean age 47.94 ± 14.39 years). No significant differences were found between groups in almost all MRDQ domains, BCVA, or EZA. Age positively correlated with 4 MRDQ domains in both groups. BCVA negatively correlated with nearly all MRDQ domains. While EZA showed a negative correlation in both groups, it was significant only in RP without CME. In the CME group, centrally located, outer nuclear layer-involving and large CS were associated with worse BCVA but better EZA.

**Conclusion:**

MRDQ responses strongly correlated with clinical parameters. CME does not seem to affect self-reported visual function in RP patients, and CS may not worsen visual function. Thus, aggressive treatment of CME in RP may not be necessary.

## Background

Retinitis pigmentosa (RP; OMIM #268000) corresponds to a group of inherited retinal disorders (IRDs) where progressive rod-cone degeneration is observed. RP is the single most frequent IRD, with an estimated prevalence of 1 in 4,000 individuals, i.e. affecting approximately 2.5 million people worldwide [1]. Recent data from our group found that IRDs affect roughly 1:3000 individuals in Portugal [2], with RP being the most frequent diagnosis among 1369 patients enrolled in the IRD-PT registry (Marta et al., submitted).

Visual symptoms in RP usually start in the second or third decades of life, with night blindness as a frequent early manifestation, followed by progressive visual field constriction [3]. Some RP hallmarks are optic disc pallor, retinal vessel attenuation, and mid-peripheral bone spicule hyperpigmentation, although the exuberance of each symptom and its impact in visual function is variable, having in mind the phenotypic heterogeneity that characterizes the disease. Reduced or non-detectable electroretinogram (ERG) responses under both scotopic and photopic conditions are common. As the disease progresses, a concentric loss of the outer retinal layers is observed, with significant visual field loss, and in advanced disease, macular atrophy with foveal involvement can be found, with severe central visual acuity loss [4, 5].

The classical notion that visual function loss in RP occurs concentrically, bilaterally and symmetrically, and that central visual acuity is preserved until late in the disease course is no longer an acquired fact. More than 90 genes contribute to the genetic and phenotypical heterogeneity that characterizes the disease [6, 7]. Early central visual acuity loss may be attributed to macular atrophy, choroidal neovascularization (CNV), vitreomacular interface disorders (VMID) and cystoid macular edema (CME). Even though the association between CME and RP has been known for a long time [8], the pathophysiology of CME in RP is still a matter of debate. The reported prevalence of CME varies in the literature but studies have shown that it may affect up to 50% of eyes [9, 10]. Optical coherence tomography (OCT) revolutionized the diagnosis and management of retinal diseases [11–13] and is currently the most frequently used imaging method to establish the presence of CME in RP [9, 14, 15]. Cystoid spaces (CS) are commonly located in the inner nuclear layer, suggesting that BRB dysfunction may be one of the main culprits [16, 17]. CME is usually observed in eyes with better vision and preserved retinal structure, although the reason for this is not fully elucidated [9].

Patient-reported outcome (PRO) measures are essential for evaluating the effectiveness of treatments and understanding how patients perceive the benefits of these treatments in a consistent manner [18, 19]. These clinical assessments reflect the patient’s perspective on how their disease affects their life, highlighting the aspects of visual impairment that influence their emotional well-being [19]. The Michigan Retinal Degeneration Questionnaire (MRDQ) [19] is a psychometrically-validated PRO measure specifically designed for patients with IRDs. It has been translated and linguistically validated for use in Portuguese-speaking countries, allowing it to capture subjective disabilities across various domains that correspond to physiological visual function pathways [20].

The purpose of this study was to evaluate self-reported visual function in RP patients with and without CME and to investigate the association between CS, retinal morphometric and clinical parameters in the CME group.

## Materials and methods

### Study design

Cross-sectional case-control study (1:3) conducted at an IRD referral center (ULS Coimbra, Portugal). The IRD-PT registry [21] was used for patient recruitment/selection. Cases included consecutive RP patients with evidence of CME (RP+CME group), while controls included age and gender-matched RP patients without CME (RP-CME group). All patients from both groups responded to the Portuguese version of the MRDQ questionnaire and underwent a thorough ophthalmological examination complemented by multimodal retinal imaging. Demographic and genetic data was obtained from each individual patient file. Informed consent was obtained for every included subject. The study was approved by the local Ethics Committee and followed the tenets of the Declaration of Helsinki for biomedical research.

### Genetic testing

Genetic testing was clinically-oriented in all probands and coordinated by a medical geneticist from the Medical Genetics Unit of ULS Coimbra. Whole exome sequencing (WES)-based next-generation sequencing (NGS) panel with copy number variant analysis was used in all probands. Peripheral blood samples were collected from all probands and available relatives for genetic analysis. The genomic DNA was extracted using a genomic DNA extraction and purification kit based on the manufacturer’s protocol. Variants were classified in accordance with the American College of Medical Genetics and Genomics (ACMG) [22]. All variants classified as pathogenic (class V) or likely pathogenic (class IV) were further confirmed by Sanger sequencing. Whenever possible, segregation analysis was performed in family members. Published cDNA sequences for the identified genes were compared with the sequencing results. Genetic counseling provided by a medical geneticist was granted to all subjects.

### Data collection and grading

Clinical and demographic data were collected from each individual patient file. All patients underwent a complete ophthalmologic examination comprising best-corrected visual acuity (ETDRS letters), anterior segment biomicroscopy, dilated fundus biomicroscopy and intraocular pressure measurement. Complementary, multimodal retinal imaging included ultra-wide field color fundus photography and fundus autofluorescence (Optos California, Optos GmbH, Germany) and swept-source OCT (Zeiss PLEX Elite 9000).

**Fig. 1:**
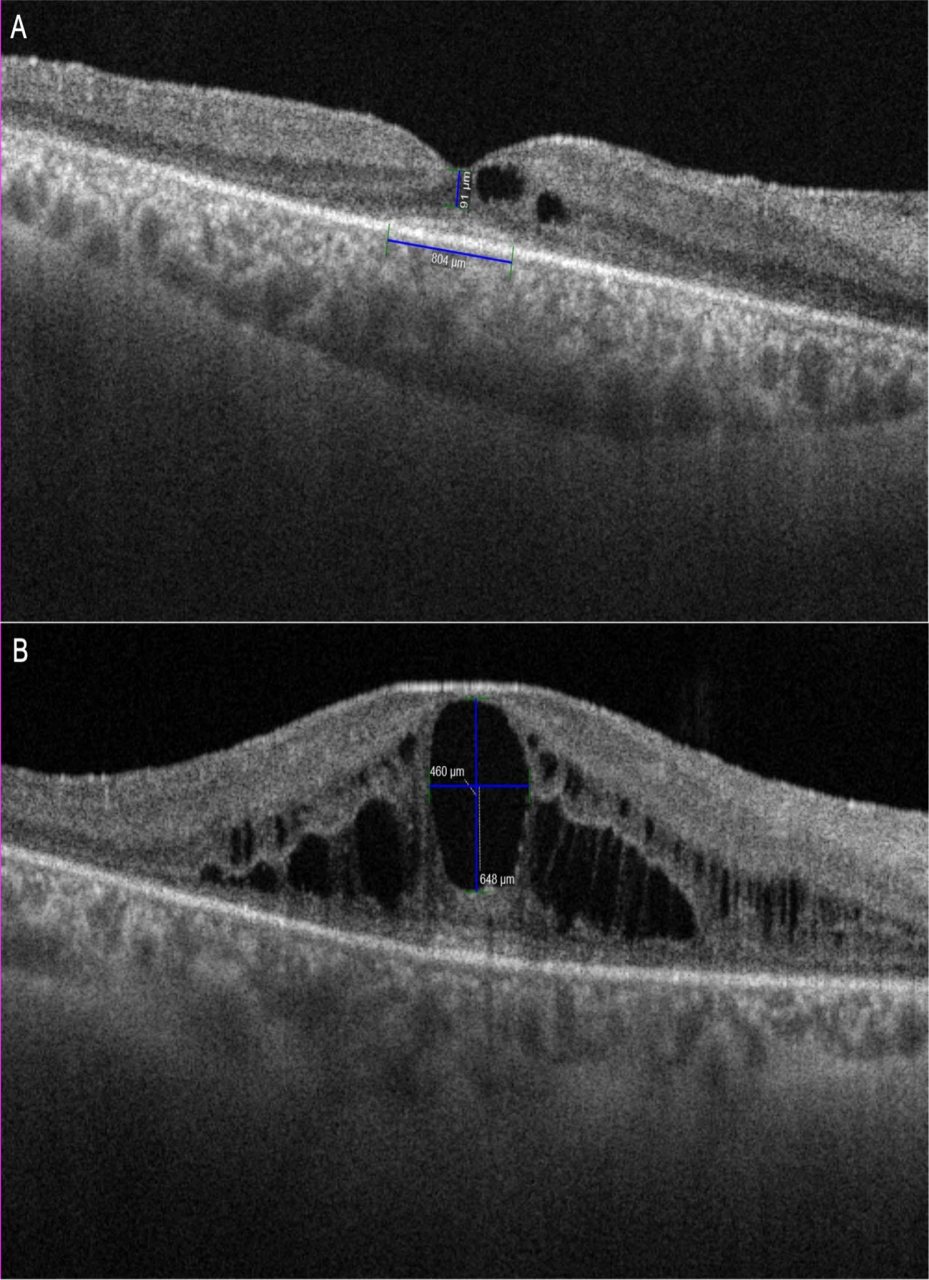
(**A**): EZ length and CPONLT measurements in horizontal scan. (**B**): CS size measurement (width and length)

**Fig. 2:**
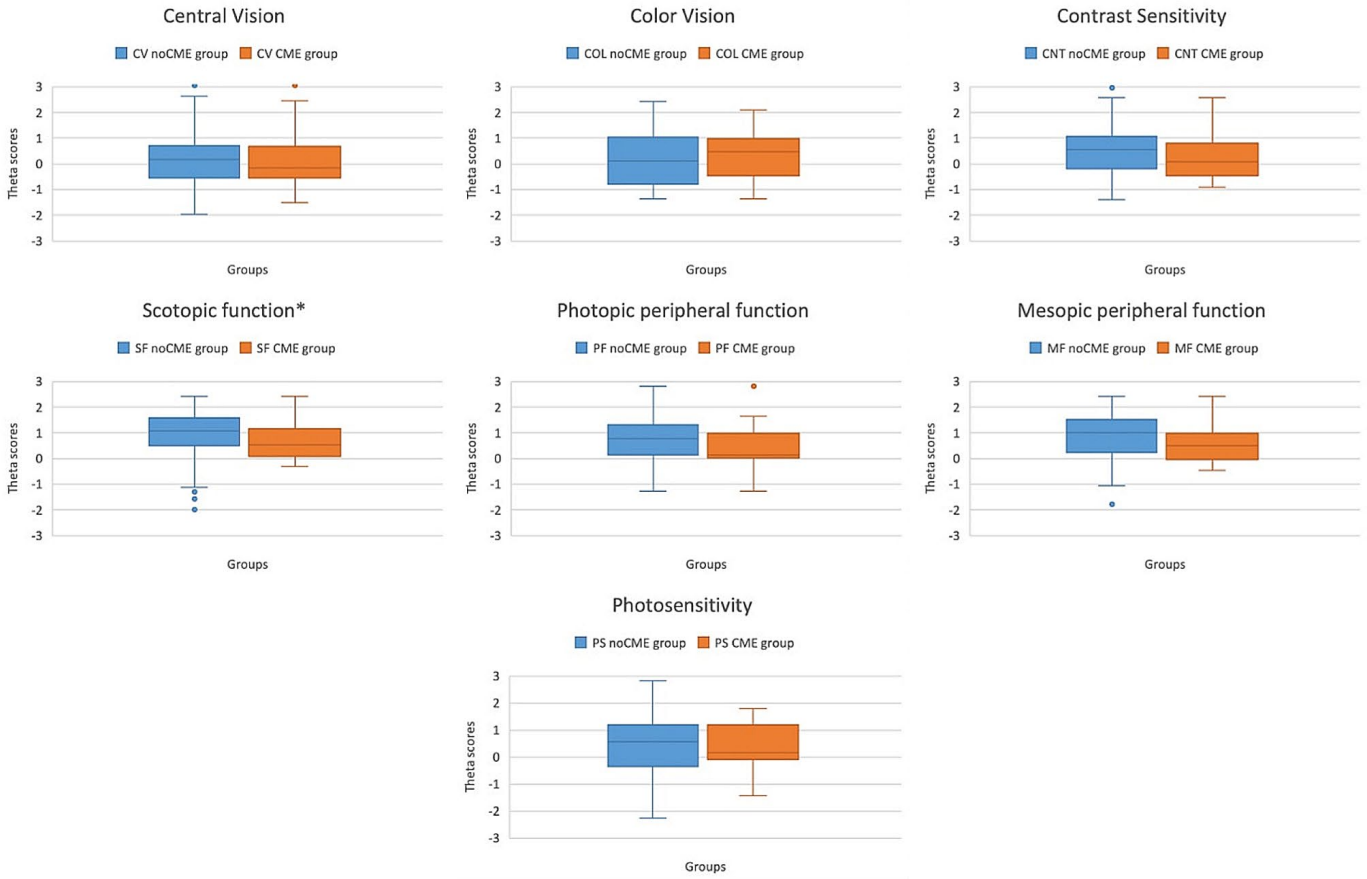
Comparison of MRDQ scores between the CME-RP group and the noCME-RP group. *indicates statistically significant difference. CNT = contrast sensitivity, COL = color vision, CV = central vision, MF = mesopic peripheral function, PF = photopic peripheral function, PS = photosensitivity, SF = scotopic function, noCME group: group without cystoid macular edema, CME group: group with cystoid macular edema

Lens status was recorded and patients classified as phakic (with or without cataract) or pseudophakic. Vitreomacular interface status was also graded, regarding vitreous attachment (attached or detached) and presence of macular hole, pseudohole, lamellar hole, epiretinal membrane (ERM) or vitreomacular traction (VMT). ERM was graded according to the ERM SD-OCT classification proposed by Govetto et al. [23]. In the RP+CME group. A morphometric assessment using the in-built software was performed by 2 independent graders (CC and CN), and included: **(1) central point outer nuclear layer thickness (CPONLT)**: manually measured in horizontal scans (Fig. 1); **(2) ellipsoid zone area (EZA)**: ellipsoid width was manually measured in horizontal and vertical scans (Fig. 1). By assuming that the EZA is a semi-oval structure, each of the vertical and horizontal widths were considered a diameter, therefore, EZA was calculated using the formula EZA = π((D1 + D2)/4)^2^; **(3) central macular thickness (CMT)**: automatically provided by the software macular thickness analysis (100 kHz)); **(4) Cystoid Space (CS) analysis**: manually measured regarding: (4.1) **location**: central point, central millimeter or outside central millimeter; (4.2) **retinal layer involvement**: inner nuclear layer or inner nuclear layer + outer nuclear layer/outer plexiform layer; **(4.3) size of the largest CS** in the central mm (Fig. 2): CS width and length were measured in axial scans and according to the greatest measure, CS were classified as: small: 0–150 μm, medium: 150–300 μm, large: 300–450 μm or very large: >450 μm.

### Michigan retinal degeneration questionnaire (MRDQ)

The MRDQ was created using item response theory, factor analysis, and graded response models [18]. It assesses how visual impairment affects everyday activities through 59 Likert-scale questions spread across seven areas: central vision, color vision, contrast sensitivity, scotopic function, photopic peripheral vision, mesopic peripheral vision, and photosensitivity [18, 19]. Item response theory analysis produces a theta score, reflecting a person’s functional ability in the tested domain. These scores are centered around zero, follow a normal distribution with a variance of 13, and range from − 3 to + 3, with higher scores indicating more severe disability [24]. All participants completed the linguistically validated Portuguese version of the MRDQ [20].

### Data analysis

The T test was used to assure that ages between groups were not significantly different. A descriptive analysis was conducted to all study variables. Data normality was visually assessed and analyzed with the Shapiro–Wilk test. Normal distributed data was presented as mean and standard deviation (SD), and non-normal distributed data was presented as median and interquartile range (IQR). BCVA was expressed in ETDRS letters. Demographic and baseline data were described according to each variable type. Continuous variables were analyzed by means of two-tailed parametric tests, such as T test or, when warranted, non-parametric tests, such as Mann Whitney test. Chi-square test was used for analysis of categorical outcome variables.

Correlations between the different parameters were tested using the Spearman correlation and Point biserial correlation coefficients. For the correlations, the BCVA of the better seeing eye and the larger EZA from the two eyes were used.

Statistically significant results were considered for p-values lower than 0.05. For statistical analysis, Microsoft Excel for mac version 16.88 (© 2024 Microsoft Corporation, USA), GraphPad Prism Software for Windows, version 8.4.2 (GraphPad Software Inc, California, USA) and SPSS version 26 (SPSS, Inc., Chicago, IL, USA) were used.

## Results

### Study population

Twenty-three patients were enrolled in the RP+CME group, while the RP-CME group had 69 patients. Mean age of the RP+CME group was 44.65 ± 13.58 (range 24–75) and 14 (60.87%) were male, while the mean age of the RP-CME group was 47.94 ± 14.39 (range 17–76) and 34 (49.28%) were male. Better-seeing eye median BCVA in ETDRS letters was 73 (IQR 65–85) in the RP+CME group and 70 (IQR: 65–80) in the RP-CME group. The median largest EZA from the two eyes of each patient was 1723.8mm^2^ (549.6-4050.6) in the CME group and 1095.8mm^2^ (209.2-4043.4) in the noCME group. Clinical and Epidemiological data, including BCVA, EZA and causal genes, are shown on Table 1.

**Table 1:** Baseline characteristics of RP+CME group, RP-CME group and all patients

Demographics				
Variable	All patients (*n* = 92)	RP+CME (*n* = 23)	RP-CME (*n* = 69)	Test score and *p* value*
Age (mean ± SD, range)	47.12 ± 14.26, 17–76	44.65 ± 13.58, 24–75	47.94 ± 14.39, 17–76	t = 0.953, *p* = 0.343
Gender	Male = 48 (52.17%) Female = 44 (47.83%)	Male = 14 (60.87%) Female = 9 (39.13%)	Male = 34 (49.28%) Female = 35 (50.72%)	𝞦²=0.929, *p* = 0.335
Better-seeing eye BCVA, ETDRS letters (median, IQR)	71 (65–81)	73 (65–85)	70 (65–80)	Z=-1.100, *p* = 0.271
EZA, median, (IQR) mm^2^	0.802 (0.0674-3.010)	1.724 (0.550–4.051)	1.096 (0.209–4.043)	Z=-1.136, *p* = 0.254
Causal gene mutation, n	*EYS =* 46 *RPGR =* 13 *CNGB1 =* 4 *RHO =* 4 *CEP290 =* 2 *RPE65 =* 2 *USH2A =* 2 *IMPG1 =* 2 *PRPF31 =* 2 *SLC66A1 =* 1 *HGSNAT =* 1 *PDE6B =* 1 *GRK1 =* 1 *ARL2BP =* 1 *AGBL5 =* 1 *NR2E3 =* 1 *PRPF8 =* 1 Inconclusive = 7	*EYS =* 9 *RHO =* 2 *RPGR =* 1 *SLC66A1 =* 1 *USH2A =* 1 *CNGB1 =* 1 *HGSNAT =* 1 *IMPG1 =* 1 *PDE6B =* 1 *PRPF31 =* 1 *Inconclusive =* 4	*EYS =* 37 *RPGR =* 12 *CNGB1 =* 3 *CEP290 =* 2 *RPE65 =* 2 *RHO =* 2 *USH2A =* 1 *IMPG1 =* 1 *PRPF31 =* 1 *GRK1 =* 1 *ARL2BP =* 1 *AGBL5 =* 1 *NR2E3 =* 1 *PRPF8 =* 1 Inconclusive = 3	NA

*Between RP + CME and RP-CME groups

NA: Not applicable

𝞦²: Chi square test value

t = Student’s t test value

Z = Mann Whitney U test score value

### Correlation between MRDQ domains and clinical parameters in the RP+CME group, RP-CME group and all patients

Gender did not correlate with any of the MRDQ domains.

Considering the whole cohort, a significant positive correlation was observed between age and all MRDQ domains apart from color vision, highlighting an increased disability with age. On the other hand, a significant negative correlation was observed between BCVA and all MRDQ domains, showing that the lower the BCVA ETDRS score, the higher the disability. A significant negative correlation was also established between EZA and all MRDQ domains, meaning that larger EZA are associated with less disability.

Correlations between MRDQ domains and gender, age, BCVA and EZA across both groups (RP+CME and RP-CME) are shown in Table 2.

**Table 2:** Spearman’s rank correlation coefficient analyses between the 7 MRDQ domains and age, gender, BCVA and EZA across the whole cohort and each individual group

MRDQ domain							
	Central vision	Color vision	Contrast sensitivity	Scotopic function	Photopic peripheral function	Mesopic peripheral function	Photosensitivity
**Whole cohort**							
Gender*	*r*=-0.107 *p* = 0.308	*r*=-0.0929 *p* = 0.379	*r*=-0.0930 *p* = 0.378	*r*=-0.0148 *p* = 0.889	*r*=-0.0799 *p* = 0.449	*r* = 0.00492 *p* = 0.963	*r* = 0.0328 *p* = 0.756
Age	*r* = 0.349 *p* = 0.00066	*r* = 0.146 *p* = 0.166	*r* = 0.264 *p* = 0.0111	*r* = 0.358 *p* = 0.00046	*r* = 0.282 *p* = 0.00652	*r* = 0.270 *p* = 0.00925	*r* = 0.250 *p* = 0.0165
BCVA	*r*=-0.673 *p* < 0.001	*r*=-0.541 *p* < 0.001	*r*=-0.551 *p* < 0.001	*r*=-0.524 *p* < 0.001	*r*=-0.532 *p* < 0.001	*r*=-0.526 *p* < 0.001	*r*=-0.374 *p* = 0.00024
EZA	*r*=-0.460 *p* < 0.001	*r*=-0.488 *p* < 0.001	*r*=-0.511 *p* < 0.001	*r*=-0.423 *p* < 0.001	*r*=-0.440 *p* < 0.001	*r*=-0.410 *p* < 0.001	*r*=-0.360 *p* = 0.00042
**RP + CME group**							
Gender*	*r*=-0.270 *p* = 0.212	*r*=-0.374 *p* = 0.0786	*r*=-0.283 *p* = 0.190	*r*=-0.345 *p* = 0.106	*r*=-0.355 *p* = 0.0961	*r*=-0.283 *p* = 0.192	*r*=-0.156 *p* = 0.477
Age	*r* = 0.527 *p* = 0.00971	*r* = 0.434 *p* = 0.0387	*r* = 0.398 *p* = 0.0601	*r* = 0.337 *p* = 0.116	*r* = 0.610 *p* = 0.00202	*r* = 0.253 *p* = 0.245	*r* = 0.674 *p* = 0.00042
BCVA	*r*=-0.710 *p* = 0.00015	*r*=-0.621 *p* = 0.00158	*r*=-0.382 *p* = 0.0720	*r*=-0.486 *p* = 0.0188	*r*=-0.505 *p* = 0.0141	*r*=-0.489 *p* = 0.0180	*r*=-0.445 *p* = 0.0334
EZA	*r*=-0.0949 *p* = 0.667	*r*=-0.147 *p* = 0.504	*r*=-0.0361 *p* = 0.870	*r*=-0.14 *p* = 0.508	*r*=-0.144 *p* = 0.511	*r*=-0.0771 *p* = 0.727	*r*=-0.244 *p* = 0.261
**RP-CME group**							
Gender*	*r*=-0.126 *p* = 0.302	*r*=-0.0393 *p* = 0.749	*r*=-0.0910 *p* = 0.457	*r*=-0.0314 *p* = 0.798	*r*=-0.0982 *p* = 0.422	*r* = 0.00473 *p* = 0.969	*r* = 0.105 *p* = 0.389
Age	*r* = 0.304 *p* = 0.0111	*r* = 0.107 *p* = 0.382	*r* = 0.243 *p* = 0.0445	*r* = 0.379 *p* = 0.0013	*r* = 0.216, *p* = 0.0746	*r* = 0.281 *p* = 0.0193	*r* = 0.136 *p* = 0.266
BCVA	*r*=-0.656 *p* < 0.001	*r*=-0.536 *p* < 0.001	*r*=-0.584 *p* < 0.001	*r*=-0.524 *p* < 0.001	*r*=-0.535 *p* < 0.001	*r*=-0.501 *p* < 0.001	*r*=-0.362 *p* = 0.00222
EZA	*r*=-0.548 *p* < 0.001	*r*=-0.569 *p* < 0.001	*r*=-0.606 *p* < 0.001	*r*=-0.468 *p* < 0.001	*r*=-0.514 *p* < 0.001	*r*=-0.451 *p* < 0.001	*r*=-0.383 *p* = 0.00117

*Point biserial correlation was used where Male = 0, Female = 1

### Comparison of MRDQ scores between the RP+CME and the RP-CME groups

Scores in the RP+CME group were overall lower in all domains, except for color vision. However, only scotopic function reached statistical significance [0.541 (0.0807–1.165) vs. 1.0568 (0.487–1.576), Z = 2.362, *p* = 0.0183]. Table 3 shows the complete data and Fig. 2 illustrates the comparison of the MRDQ scores between the two groups.

**Table 3:** Comparison of the RP+CME group and RP-CME group MRDQ scores

Demographics				
Variable	All patients (*n* = 92)	RP+CME (*n* = 23)	RP-CME (*n* = 69)	Test score and *p* value*
MRDQ theta scores (median, IQR)				
Central vision	0.143 (-0.546-0.695)	-0.157 (-0.540-0.670)	0.170(-0.560-0.717)	*Z =* 0.442 *p* = 0.660
Color vision	0.269 (-0.693-1.007)	0.479 (-0.464-0.975)	0.0956(-0.782-1.0360)	*Z=*-0.415 *p* = 0.682
Contrast sensitivity	0.478 (-0.227-1.046)	0.0762 (-0.454-0.796)	0.548 (-0.185-1.0787)	Z = 1.064 *p* = 0.289
Scotopic function	1.019 (0.330–1.516)	0.541 (0.0807–1.165)	1.0568 (0.487–1.576)	**Z = 2.362** *p* = 0.0183
Photopic peripheral function	0.678 (0.0715–1.269)	0.152 (0.0116–0.993)	0.766 (0.134–1.323)	Z = 1.830 *p* = 0.0672
Mesopic peripheral function	0.809 (0.170–1.487)	0.496 (-0.0415-0.982)	0.999 (0.219–1.511)	Z = 1.650 *p* = 0.0989
Photosensitivity	0.496 (-0.223-1.185)	0.165 (-0.0937-1.190)	0.548 (-0.342-1.187)	Z = 0.140 *p* = 0.889

* Between RP + CME and RP-CME groups

Z = Mann Whitney U test score value

### Characterization of the RP+CME group

Table 4 presents a detailed characterization of the RP+CME group, including morphometric parameters.

**Table 4:** Baseline characteristics of the RP+CME group and morphometric parameters

**Characteristics of the study population**		
Patients (n)		23
Eyes (n)		41
Gender (n,%)		
Male		14 (60.87%)
Female		9 (39.13%)
Age (mean ± SD)		44.65 ± 13.58 years
BCVA (median, interquartile range)		
logMAR		(0.3, 0.02–0.42)
ETDRS letters		(70, 65–84)
Inheritance pattern (n,%)		
AR		15 (65.22%)
AD		3 (13.04%)
XL		1 (4.35%)
Simplex cases		2 (8.70%)
Genetically-unsolved cases		2 (8.70%)
Non-Syndromic RP (n,%)		21 (91.30%)
Syndromic RP (n,%)		2 (8.70%)
Lens status (n,%)		
Phakic		34 (82.93%)
Clear lens		22 (64.71%)
Cataract (all posterior subcapsular)		12 (35.29%)
Pseudophakic		7 (17.07%)
Vitreomacular interface status (n,%)		
Bilateral CME (patients)		18 (78.26%)
ERM (eyes)		10 (24.39%)
PVD (eyes)		4 (9.76%)
VMT (eyes)		1 (2.44%)
MH, LH or PMH (eyes)		0 (0%)
**Morphometric parameters**		
CS location		
CP	15 eyes (36.59%)	
CM	19 (46.34%)	
OCM	7 eyes (17.07%)	
CS retinal layer involvement		
INL	19 eyes (46.34%)	
INL + OPL/ONL	15 eyes (36.59%)	
INL + GCL	3 eyes (7.32%)	
INL + OPL/ONL + GCL	4 eyes (9.76%)	
CS size		
Very large	8 (19.51%)	
Large	7 (17.07%)	
Medium	9 (21.95%)	
Small	17 (41.46%)	
Mean CMT	0.296 (0.263–0.348) mm	
Mean CPONLT	0.102 (0.083–0.150) mm	
Mean EZA width and area		
Horizontal scan	1.732 (0.951–2.494) mm	
Vertical scan	0.927 (0.602–1.625) mm	
Area	1.254 (0.520–2.932) mm^2^	

SD: standard deviation; BCVA: best-corrected visual acuity; AR: Autosomal-recessive; AD: autosomal-dominant; XL: X-linked; RP: retinitis pigmentosa; CME: cystoid macular edema; ERM: epiretinal membrane; PVD: posterior vitreous detachment; VMT: vitreomacular traction; MH: macular hole; LH: lamellar hole; PMH: pseudomacular hole; CS: cystoid spaces; CP: central point; CM: central millimeter; OCM: out of central millimeter; INL: inner nuclear layer; OPL/ONL: outer plexiform layer/outer nuclear layer; GCL: ganglion cell layer; CMT: central macular thickness; CPONLT: central point outer nuclear layer thickness; EZA: ellipsoid zone area

In 8 eyes − 19.51% (all with CS in the CP), the central point location of the CS precluded the measurement of the CPONLT, so this item in these patients was further classified as non-measurable.

### Associations between CS parameters (retinal layer involvement, size and location), BCVA and EZA

Retinal layer involvement and CS size did not impact EZA or BCVA. Regarding CS location, patients with CP CS showed better BCVAs (Z = 2.192, *p* = 0.0285), but smaller EZA (Z=-0.936, *p* = 0.347). Finally, BCVA positively correlated with CMT and with EZA (Table 5).

**Table 5:** Relationship between CME morphometric parameters and BCVA/ EZA

1) Retinal layers involvement			
Anatomical structure or clinical feature	CS in INL	CS in INL + ONL	Z score* and p value
EZA (median, IQR) mm^2^	1.143 (0.518–3.104)	1.622 (0.474–2.920)	Z=-0.219 *p* = 0.826
BCVA (median, IQR) ETDRS letters	75 (65–85)	70 (65–80)	Z = 1.103 *p* = 0.271

2) CS size			
Anatomical structure or clinical feature	Small and medium CS	Large and very large CS	Z score* and p value
EZA (median, IQR) mm^2^	1.143 (0.523–2.730)	1.840 (0.303–3.577)	Z=-0.621 *p* = 0.535
BCVA (median, IQR) ETDRS letters	75 (65–85)	70 (63–75)	Z = 1.375 *p* = 0.171

3) CS location			
Anatomical structure or clinical feature	CS in CP	CS in CM and OCM	z score* and p value
CMT (mm), median (IQR)	0.449 (0.323–0.564)	0.269 (0.246–0.293)	Z=-3.969, *p* = 0.0008
EZA (mm^2^), median (IQR)	1.716 (0.758–4.051)	1.139 (0.518–2.871)	Z=-0.936, *p* = 0.347
BCVA, median (IQR)	70 (63–75)	81 (65–85)	Z = 2.192, *p* = 0.0285

4) CPONLT and CMT			
Anatomical structure or clinical feature	Spearman correlation coefficient and p value		
	CPONLT	CMT	BCVA
EZA	*r* = 0.2034, *p* = 0.255	*r* = 0.0324 *p* = 0.847	*r* = 0.381 *p* = 0.0154
BCVA	*r*=-0.0824 *p* = 0.649	*r*=-0.402 *p* = 0.0112	NA

*Mann-Whitney U test

BCVA: best-corrected visual acuity; CS: cystoid spaces; CP: central point; CM: central millimeter; OCM: out of central millimeter; CMT: central macular thickness; INL: inner nuclear layer; ONL: outer nuclear layer; EZA: ellipsoid zone area; CPONLT: central point outer nuclear layer thickness; NA: not applicable

### Interdependence between CS parameters

Patients with CS in the CP had CS located mostly in the INL + ONL, while patients with CS in CM and OCM had CS located mostly in the INL. Patients with CS in the CP had CS sized large or very large, while patients with CS in CM and OCM had CS sized small and medium. Large and very large CS were located mostly in the INL + ONL, while small and medium CS were located mostly in the INL (Table 6).

**Table 6:** Association of the interdependence between CS location, CS retinal involvement and CS size

CS location and CS retinal involvement			Chi square value and *p* value
	INL	INL + ONL	𝞦²=8.319 *p* = 0.0039
CP	4	11	
CM and OCM	19	7	

CS location and CS size			Chi square value and p value
	Large and very large	Small and medium	𝞦²=13.768 *p* = 0.0002
CP	11	4	
CM and OCM	4	22	

CS retinal layer involvement and CS size			Chi square value and p value
	Large and very large	Small and medium	𝞦²=8.319 *p* = 0.0039
INL	4	19	
INL + ONL	11	7	

*Chi-square test

CS: cystoid spaces; CP: central point; CM: central millimeter; OCM: out of central millimeter; INL: inner nuclear layer; ONL: outer nuclear layer; EZA: ellipsoid zone area

## Discussion

Self-reported health status and patient’s perception of visual function has been found to correlate more closely with quality of life than conventional tests [25] However, in the specific case of IRDs, patients’ voices used to be underrepresented due to a lack of reliable PRO measures [26]. The MRDQ [19] is a psychometrically validated PRO instrument specifically designed to assess visual function in IRDs that has been shown to correlate with clinician reported outcomes across several RP-associated genes [18, 24, 27, 28]. The association of CME and RP is well established but the real impact of RP-associated CME in these patients’ visual function has never been investigated. In this case-control study, we aimed to evaluate self-reported visual function in RP patients with and without CME and to further investigate the association between CS, retinal morphometric parameters and clinical data using SS-OCT.

First, the whole cohort analysis showed strong correlations between MRDQ domains and age, BCVA and EZA, thus confirming that in RP patients increased disability occurs with increasing age, lower BCVA and smaller EZA. This is in agreement with previous studies [18, 24, 27, 28] and supports the use of MRDQ in RP.

Second, we compared self-reported visual function between RP patients with and without CME and despite overall lower MRDQ scores in the CME group, the only statistically significant difference observed was in scotopic function. Scotopic function is dependent on rod photoreceptor impairment which primarily happens in the retinal periphery and not in the center, where CME occurs. We hypothesize that this difference is largely due to the genotype heterogeneity both within and between groups. Theoretically, the MRDQ domains that could be negatively impacted by CME would be central vision, color vision, contrast sensitivity and photosensitivity. Interestingly, differences between groups were not significant in any of these domains. Moreover, a strong correlation was found between BCVA and MRDQ domains, irrespective of the presence of CME. This may explain why previous studies by Arrigo et al. [29] and Yoon Jeon Kim et al. [30], did not find significant differences in BCVA between eyes with and without CME. Our findings point towards a minimal impact of CME in patient’s visual function, thus raising an important question: should we treat RP-associated CME? Overall, tolerance to fluid accumulation in the retina (especially intra-retinal cysts) is usually very low, largely due to our experience in diabetic macular edema and neovascular age-related macular degeneration [31–35]. The mechanisms behind fluid accumulation in these conditions include microvascular damage and leakage, ischemia, acute inflammation with release of prostaglandins and cytokines, or abnormal vessels growth [36–39], and clinical trials have shown that blockage of these pathways are an effective treatment. However, the pathogenesis of CME in RP remains incompletely understood and even though several strategies to treat RP-associated CME have been tried [40–45], the results differ greatly among studies and no specific treatment guidelines are available. Lastly, a significant negative correlation was also established between EZA and all MRDQ domains in the RP-CME group, but not in the RP + CME group, which showed a weak not significant negative correlation. We hypothesize that EZA is a poor marker of visual function due to difficulties determining EZA’s in the presence of CS (shadow effect).

Third, we investigated the association between CS, retinal morphometric parameters and clinical data using SS-OCT. Retinal layer involvement and CS size did not impact EZA or BCVA. This contrasts with the impact of CME seen in patients with diabetic macular edema and branch retinal vein occlusion [46], thus pointing towards different mechanisms in RP-associated CME. One interesting finding was that patients with centrally located CS showed larger EZA, thus suggesting a protective role of CS to the outer retina. Once again, this underscores the controversy of exhaustive efforts to completely resolve RP-associated CME. Conflicting evidence exists about the impact of CME in the inner and outer retina [29, 47]. Despite previous evidence establishing CPONLT as a biomarker of central visual function, we did not find a significant association between CPONLT and EZA or BCVA. We hypothesize that in the presence of CME, CPONLT is not a reliable biomarker of visual function since cystoid spaces may falsely increase its thickness. This is in line with the findings of Ruff et al. [47] where the authors found a correlation between the outer retinal layer area and CS size but no correlation with BCVA.

Breakdown of the blood-retinal barrier (BRB), dysfunction of the RPE pump, and Müller cell impairment have been suggested as possible etiologies of CME in RP [48]. In our study, patients with centrally-located CS had worse BCVAs but better EZAs. This might mean that CS help protect and preserve the outer retina, even though they can interfere with central vision, which per se is a very limited measure of visual function. Additionally, most CS in the CP were found in both the INL and ONL, rather than just in the INL. These CS were mostly large or very large. This suggests a protective effect, as CS closer to the outer retina avoid damaging the INL. The compression effect is spread across both inner and outer retina layers, so Müller cells are less affected than if the largest CS were only in the inner layers.

Despite the novelty, this study is not exempt of limitations. First, the RP + CME group is relatively small, which limits the strength of our conclusions. Future research with larger cohorts is needed to gain a deeper understanding of this condition and its clinical impact on visual function. Second, our study lacks long-term follow-up, which would have provided insight into temporal changes. Third, the associations and correlations we observed may not necessarily imply direct causation, given the complexity of RP-CME. Fourth, we did not account for some potentially confounding factors, such as medication or systemic conditions.

In conclusion, in this case-control study we showed that self-perceived visual function in RP does not seem to be affected by the presence of CME. Thus, efforts to successfully treat CME may be unnecessary. Gaining a deeper understanding of the mechanisms driving CME in RP is needed in order to pave the way for more judicious and evidence-based management decisions.

## Data Availability

No datasets were generated or analysed during the current study.

## Declarations

### Protection of human and animal subjects

The authors declare that the procedures followed were in accord-ance with the regulations of the relevant clinical research ethics committee and with those of the Code of Ethics of the World Medical Association (Declaration of Helsinki as revised in 2013).

### Provenance and peer review

Not commissioned; externally peer reviewed.

## References

[1] Dias MF, Joo K, Kemp JA, Fialho SL, da Silva Cunha A Jr, Woo SJ, et al. Molecular genetics and emerging therapies for retinitis pigmentosa: basic research and clinical perspectives. Prog Retin Eye Res. 2018;63:107–31. 29097191 10.1016/j.preteyeres.2017.10.004

[2] Marques JP, Ferreira N, Moreno N, Marta A, Vaz-Pereira S, Estrela-Silva S, et al. Current management of inherited retinal degenerations in Portugal (IRD-PT survey). Sci Rep. 2024;14:21473. 39277603 10.1038/s41598-024-72589-4 PMC11401845

[3] Santos T, Warren LH, Santos AR, Marques IP, Kubach S, Mendes LG, et al. Swept-source OCTA quantification of capillary closure predicts ETDRS severity staging of NPDR. Br J Ophthalmol. 2022;106:712–8. 33355147 10.1136/bjophthalmol-2020-317890 PMC9046755

[4] Ogino K, Oishi A, Oishi M, Gotoh N, Morooka S, Sugahara M, et al. Efficacy of column scatter plots for presenting retinitis pigmentosa phenotypes in a Japanese cohort. Transl Vis Sci Technol. 2016;5:4. 26966640 10.1167/tvst.5.2.4 PMC4782824

[5] Sorrentino FS, Gallenga CE, Bonifazzi C, Perri P. A challenge to the striking genotypic heterogeneity of retinitis pigmentosa: a better understanding of the pathophysiology using the newest genetic strategies. Eye. 2016;30:1542–8. 27564722 10.1038/eye.2016.197 PMC5177762

[6] Hartong DT, Berson EL, Dryja TP. Retinitis pigmentosa. Lancet. 2006;368:1795–809. 17113430 10.1016/S0140-6736(06)69740-7

[7] Nguyen X-T-A, Moekotte L, Plomp AS, Bergen AA, van Genderen MM, Boon CJF. Retinitis pigmentosa: current clinical management and emerging therapies. Int J Mol Sci. 2023;24. 10.3390/ijms24087481. 10.3390/ijms24087481 PMC10139437 37108642

[8] Fishman GA, Maggiano JM, Fishman M. Foveal lesions seen in retinitis pigmentosa. Arch Ophthalmol. 1977;95:1993–6. 921578 10.1001/archopht.1977.04450110087008

[9] Marques JP, Neves E, Geada S, Carvalho AL, Murta J, Saraiva J, et al. Frequency of cystoid macular edema and vitreomacular interface disorders in genetically solved syndromic and non-syndromic retinitis pigmentosa. Graefes Arch Clin Exp Ophthalmol. 2022;260:2859–66. 35389060 10.1007/s00417-022-05649-y

[10] Chen C, Liu X, Peng X. Management of cystoid macular edema in retinitis pigmentosa: a systematic review and meta-analysis. Front Med. 2022;9:895208. 10.3389/fmed.2022.895208 PMC9149278 35652079

[11] Kal M, Brzdęk M, Winiarczyk M, Mackiewicz J, Kozieł D, Odrobina D, et al. Retinal thickness in patients with elevated D-dimer and interleukin-6 levels as a result of SARS-CoV-2 infection. Med Stud. 2023;39:342–51.

[12] Kal M, Płatkowska-Adamska B, Zarębska-Michaluk D, Rzymski P. Reduced vessel density and enlarged foveal avascular zone in the macula as a result of systemic hypoxia caused by SARS-CoV-2 infection. J Pers Med. 2023;13. 10.3390/jpm13060926. 10.3390/jpm13060926 PMC10301350 37373915

[13] Lee J-Y, Kim JP, Jang H, Kim J, Kang SH, Kim JS, et al. Optical coherence tomography angiography as a potential screening tool for cerebral small vessel diseases. Alzheimers Res Ther. 2020;12:73. 32527301 10.1186/s13195-020-00638-x PMC7291486

[14] Yeo JH, Kim YJ, Yoon YH, Optical coherence tomography, angiography in patients with retinitis pigmentosa-associated cystoid macular Edema. Retina. 2020;40:2385–95. 31923123 10.1097/IAE.0000000000002756

[15] Gorovoy IR, Gallagher DS, Eller AW, Mayercik VA, Friberg TR, Schuman JS. Cystoid macular edema in retinitis pigmentosa patients without associated macular thickening. Semin Ophthalmol. 2013;28:79–83. 23448561 10.3109/08820538.2012.760614 PMC5536830

[16] Strong SA, Hirji N, Quartilho A, Kalitzeos A, Michaelides M. Retrospective cohort study exploring whether an association exists between spatial distribution of cystoid spaces in cystoid macular oedema secondary to retinitis pigmentosa and response to treatment with carbonic anhydrase inhibitors. Br J Ophthalmol. 2019;103:233–7. 29706600 10.1136/bjophthalmol-2017-311392

[17] Strong S, Liew G, Michaelides M. Retinitis pigmentosa-associated cystoid macular oedema: pathogenesis and avenues of intervention. Br J Ophthalmol. 2017;101:31–7. 27913439 10.1136/bjophthalmol-2016-309376 PMC5256121

[18] Karuntu JS, Nguyen XT, Boon CJF. Correlations between the Michigan Retinal Degeneration Questionnaire and visual function parameters in patients with retinitis pigmentosa. Acta Ophthalmol. 2024;102:555–63. 38158751 10.1111/aos.16601

[19] Lacy GD, Abalem MF, Andrews CA, Popova LT, Santos EP, Yu G, et al. The Michigan Retinal Degeneration Questionnaire: a patient-reported outcome instrument for inherited retinal degenerations. Am J Ophthalmol. 2021;222:60–8. 32858027 10.1016/j.ajo.2020.08.032 PMC7907279

[20] Marques JP, Bernardes L, Oliveira C, Fonseca G, Quadrado Gil J, Sotero L, et al. Portuguese translation and linguistic validation of the Michigan Retinal Degeneration Questionnaire and the Michigan Vision-related anxiety questionnaire in a cohort with inherited retinal degenerations. Ophthalmic Genet. 2022;43:137–9. 35021937 10.1080/13816810.2022.2025609

[21] Marques JP, Carvalho AL, Henriques J, Murta JN, Saraiva J, Silva R. Design, development and deployment of a web-based interoperable registry for inherited retinal dystrophies in Portugal: the IRD-PT. Orphanet J Rare Dis. 2020;15:304. 33109251 10.1186/s13023-020-01591-6 PMC7590677

[22] Richards S, Aziz N, Bale S, Bick D, Das S, Gastier-Foster J, et al. Standards and guidelines for the interpretation of sequence variants: a joint consensus recommendation of the American College of Medical Genetics and Genomics and the Association for Molecular Pathology. Genet Med. 2015;17:405–24. 25741868 10.1038/gim.2015.30 PMC4544753

[23] Govetto A, Lalane RA, Sarraf D, Figueroa MS, Hubschman JP. Insights into epiretinal membranes: presence of ectopic inner foveal layers and a new optical coherence tomography staging scheme. Am J Ophthalmol. 2017;175:99–113. 27993592 10.1016/j.ajo.2016.12.006

[24] Marques JP, Machado Soares R, Simão S, Abuzaitoun R, Andrews C, Alves CH, et al. Self-reported visual function and psychosocial impact of visual loss in EYS-associated retinal degeneration in a Portuguese population. Ophthalmic Genet. 2023;44:334–40. 36946380 10.1080/13816810.2023.2191708

[25] Bambara JK, Wadley V, Owsley C, Martin RC, Porter C, Dreer LE. Family functioning and low vision: a systematic review. J Vis Impair Blind. 2009;103:137–49. 20046836 PMC2798155

[26] Leroy BP, Daly A, Héon E, Sahel J-A, Dollfus H, IRD Study Group. Therapies for inherited retinal dystrophies: what is enough? Drug Discov Today. 2024;29:104095. 38992419 10.1016/j.drudis.2024.104095

[27] Gouveia N, Chukwunalu O, Oliveira C, Alves CH, Silva R, Murta J, et al. Exploring self-reported visual function and vision-related anxiety in patients with RPGR-associated retinal degeneration. Sci Rep. 2024;14:15189. 38956231 10.1038/s41598-024-66170-2 PMC11220147

[28] Parekh B, Duncan JL, Samarakoon L, Melia M, Abalem MF, Andrews CA, et al. Self-reported functional vision in USH2A-associated retinal degeneration as measured by the Michigan Retinal Degeneration Questionnaire. Invest Ophthalmol Vis Sci. 2024;65:5. 38833260 10.1167/iovs.65.6.5 PMC11156206

[29] Arrigo A, Aragona E, Perra C, Bianco L, Antropoli A, Saladino A, et al. Characterizing macular edema in retinitis pigmentosa through a combined structural and microvascular optical coherence tomography investigation. Sci Rep. 2023;13:800. 36646739 10.1038/s41598-023-27994-6 PMC9842653

[30] Kim YJ, Joe SG, Lee D-H, Lee JY, Kim J-G, Yoon YH. Correlations between spectral-domain OCT measurements and visual acuity in cystoid macular edema associated with retinitis pigmentosa. Invest Ophthalmol Vis Sci. 2013;54:1303–9. 23329664 10.1167/iovs.12-10149

[31] Eichenbaum D, Brown DM, Ip M, Khanani AM, Figueroa MS, McAllister IL, et al. Impact of retinal fluid-free months on outcomes in neovascular age-related macular degeneration: a treatment agnostic analysis of the HAWK and HARRIER studies. Retina. 2023;43:632–40. 36705252 10.1097/IAE.0000000000003699 PMC10035657

[32] Zur D, Guymer R, Korobelnik J-F, Wu L, Viola F, Eter N et al. Impact of residual retinal fluid on treatment outcomes in neovascular age-related macular degeneration. Br J Ophthalmol. 2024:bjo-2024-325640. 10.1136/bjo-2024-325640 10.1136/bjo-2024-325640 PMC11866303 39033013

[33] Kim JH, Sagong M, Woo SJ, Kim YC, Cho H, Lee YH, et al. A real-world study assessing the impact of retinal fluid on visual acuity outcomes in patients with neovascular age-related macular degeneration in Korea. Sci Rep. 2022;12:14166. 35986074 10.1038/s41598-022-18158-z PMC9391430

[34] Kalur A, Iyer AI, Muste JC, Talcott KE, Singh RP. Impact of retinal fluid in patients with diabetic macular edema treated with anti-VEGF in routine clinical practice. Can J Ophthalmol. 2023;58:271–7. 35395214 10.1016/j.jcjo.2022.03.003

[35] Ehlers JP, Uchida A, Sevgi DD, Hu M, Reed K, Berliner A, et al. Retinal fluid volatility associated with interval tolerance and visual outcomes in diabetic macular edema in the VISTA phase III trial. Am J Ophthalmol. 2021;224:217–27. 33253664 10.1016/j.ajo.2020.11.010 PMC9307417

[36] Stitt AW, Curtis TM, Chen M, Medina RJ, McKay GJ, Jenkins A, et al. The progress in understanding and treatment of diabetic retinopathy. Prog Retin Eye Res. 2016;51:156–86. 26297071 10.1016/j.preteyeres.2015.08.001

[37] Bélair M-L, Kim SJ, Thorne JE, Dunn JP, Kedhar SR, Brown DM, et al. Incidence of cystoid macular edema after cataract surgery in patients with and without uveitis using optical coherence tomography. Am J Ophthalmol. 2009;148:128–e352. 19403110 10.1016/j.ajo.2009.02.029 PMC2722753

[38] Kim SJ, Belair M-L, Bressler NM, Dunn JP, Thorne JE, Kedhar SR, et al. A method of reporting macular edema after cataract surgery using optical coherence tomography. Retina. 2008;28:870–6. 18536605 10.1097/IAE.0b013e318169d04e

[39] Grossniklaus HE, Green WR. Choroidal neovascularization. Am J Ophthalmol. 2004;137:496–503. 15013874 10.1016/j.ajo.2003.09.042

[40] Bakthavatchalam M, Lai FHP, Rong SS, Ng DS, Brelen ME. Treatment of cystoid macular edema secondary to retinitis pigmentosa: a systematic review. Surv Ophthalmol. 2018;63:329–39. 28987613 10.1016/j.survophthal.2017.09.009

[41] Heutinck PAT, van den Born LI, van Laar JAM, van Hagen PM, Smailhodzic D, Meester-Smoor MA et al. Somatostatin analogues as a treatment option for cystoid maculopathy in retinitis pigmentosa. BMJ Open Ophthalmol. 2024;9: e001722. 10.1136/bmjophth-2024-001722 PMC11331938 39134323

[42] Veritti D, Sarao V, De Nadai K, Chizzolini M, Parmeggiani F, Perissin L, et al. Dexamethasone implant produces better outcomes than oral acetazolamide in patients with cystoid macular edema secondary to retinitis pigmentosa. J Ocul Pharmacol Ther. 2020;36:190–7. 31886707 10.1089/jop.2018.0153

[43] Català-Mora J, Santamaría Álvarez JF, Kyriakou D, Alforja S, Barraso Rodrigo M, Blasco Palacio PB, et al. Protocol for the treatment of cystoid macular edema secondary to retinitis pigmentosa and other inherited retinal dystrophies. Arch Soc Esp Oftalmol (Engl Ed). 2024;99:67–81. 37940089 10.1016/j.oftale.2023.11.001

[44] Colombo L, Montesano G, Di Domenico A, Colizzi B, Rissotto R, Maltese P, et al. Dexamethasone implant versus topical carbonic anhydrase inhibitors in patients with bilateral retinitis pigmentosa-related cystoid macular edema: a prospective, paired-eye pilot study. Retina. 2024;44:852–60. 38166238 10.1097/IAE.0000000000004039 PMC11027988

[45] Huang Q, Chen R, Lin X, Xiang Z. Efficacy of carbonic anhydrase inhibitors in management of cystoid macular edema in retinitis pigmentosa: a meta-analysis. PLoS ONE. 2017;12: e0186180. 10.1371/journal.pone.0186180 PMC5638411 29023491

[46] Mimouni M, Nahum Y, Levant A, Levant B, Weinberger D. Cystoid macular edema: a correlation between macular volumetric parameters and visual acuity. Can J Ophthalmol. 2014;49:183–7. 24767226 10.1016/j.jcjo.2013.11.004

[47] Ruff A, Tezel A, Tezel TH. Anatomical and functional correlates of cystic macular edema in retinitis pigmentosa. PLoS ONE. 2022;17:e0276629. 36269735 10.1371/journal.pone.0276629 PMC9586413

[48] Yoshida N, Ikeda Y, Notomi S, Ishikawa K, Murakami Y, Hisatomi T, et al. Clinical evidence of sustained chronic inflammatory reaction in retinitis pigmentosa. Ophthalmology. 2013;120:100–5. 22986109 10.1016/j.ophtha.2012.07.006

